# Brittle stars looking like starfish: the first fossil record of the Astrophiuridae and a remarkable case of convergent evolution

**DOI:** 10.7717/peerj.8008

**Published:** 2019-11-13

**Authors:** Ben Thuy, Andy Gale, Lea Numberger-Thuy

**Affiliations:** 1 Department of Palaeontology, Natural History Museum Luxembourg, Luxembourg City, Luxembourg; 2 School of Earth & Environmental Sciences, University of Portsmouth, Portsmouth, UK

**Keywords:** Ophiuroidea, Astrophiuridae, Amphilimnidae, New taxa, Cretaceous, Convergent evolution, Microfossils

## Abstract

The genus *Astrophiura*, which ranks among the most extraordinary of modern brittle stars, is the type genus of the recently resurrected family Astrophiuridae within the order Ophiurida. On account of its absurdly enlarged and strongly modified lateral arm plates, *Astrophiura* bears a closer resemblance to a pentagonal starfish than to a typical ophiuroid. Although molecular evidence suggests an ancient origin of the Astrophiuridae, dating back at least to the Early Jurassic, not a single fossil astrophiurid has been reported so far. Here, we describe dissociated lateral arm plates from the Campanian of Cringleford near Norwich, UK, and the Maastrichtian of Rügen, Germany (both Upper Cretaceous) with unambiguous astrophiurid affinities and assign these to a new species, *Astrophiura markbeneckei*. This represents the first fossil record of the family. In addition, the Rügen material included lateral arm plates that superficially resemble those of *A. markbeneckei* sp. nov. but differ in having spine articulations that are typical of the ophionereidoid family Amphilimnidae. We assign these plates to a new genus and species, *Astrosombra rammsteinensis*, an extinct amphilimnid with morphological modifications similar to those of *Astrophiura*, and thus representing a remarkable case of parallel evolution amongst brittle stars looking like starfish.

## Introduction

Occasionally, scientists are struck by discoveries to such extent that they express their enthusiasm explicitly, for example by choosing a name for a new species that translates as ‘very wonderful’. This was most likely the case when British biologist Sladen (1878, 1879) introduced the species name *permira* for an odd brittle star with a bizarre, limpet-like body shape unseen in any other ophiuroid. The unusual morphology of this taxon, and the absurdly large disc with confusing plate patterns in particular, prompted Sladen (1878, 1879) to speculate on an intermediate position of this form between the brittle stars (Ophiuroidea) and the starfish (Asteroidea), which is expressed in the genus name *Astrophiura*.

More than a century of research later, *Astrophiura* is now unanimously considered to be an ophiuroid and nothing more (Koehler, 1915; Clark, 1923; Cherbonnier & Guille, 1976; Vadon, 1991; Fujita & Hendler, 2001; Pawson, 2018). The morphological peculiarities of *Astrophiura* are the result of a general reduction in body size combined with a notable enlargementof proximal lateral arm plates that form a tightly interlocking array of dorso-ventrally flattened ossicles and extend the disc well beyond its usual periphery. Sladen (1879) considered these characters to be sufficiently unique to assign *Astrophiura* to its own family, Astrophiuridae. In his revision of ophiuroid classification, however, Matsumoto (1915) did not accept the family Astrophiuridae and transferred *Astrophiura* to the ophiolepidid subfamily Ophiomastinae Matsumoto (1915), which has subsequently been synonymized with the ophiurid subfamily Ophiurinae Lyman (1865) (O’Hara et al., 2018). Later authors mostly followed Matsumoto (1915) transfer of *Astrophiura* (Spencer & Wright, 1966) to the ophiurids.

Recent advances in ophiuroid phylogeny and evolution have led to a thorough restructuring of the classification of the class Ophiuroidea (O’Hara et al., 2018). Molecular data have now confirmed the close phylogenetic ties between *Astrophiura* and the ophiurid brittle stars but at the same time have suggested separation at the family level, which has resulted in the resurrection of the Astrophiuridae (O’Hara et al., 2017). To date, this family comprises, in addition to the type genus *Astrophiura*, the genera *Ophiophycis* Koehler (1901) and *Ophiomisidium* Koehler (1914) (O’Hara et al., 2018). Although the clade is estimated to have diverged during the Early Jurassic (O’Hara et al., 2017), not a single fossil had hitherto been referred to the Astrophiuridae, making it one of the very few ophiuroid families without a definite fossil record, until now.

There are several reasons why such an ancient clade has gone unrecognised for decades. First, living astrophiurids are inconspicuous and restricted to bathyal settings and rarely sampled for that reason. Second, in the light of the extreme patchiness of the deep-sea fossil record (Thuy et al., 2012), it is even more difficult to find extinct astrophiurids. And third, to make matters worse, ophiuroids generally disintegrate rapidly after death and fossilize as dissociated microscopic plates rather than intact skeletons, especially in environments with low sedimentation rates such as the deep sea. In *Astrophiura*, the most diagnostic skeletal ossicles in particular, that is the lateral arm plates, are strongly modified. These are insufficiently illustrated in most descriptions, hampering recognition by non-experts.

**Figure 1:**
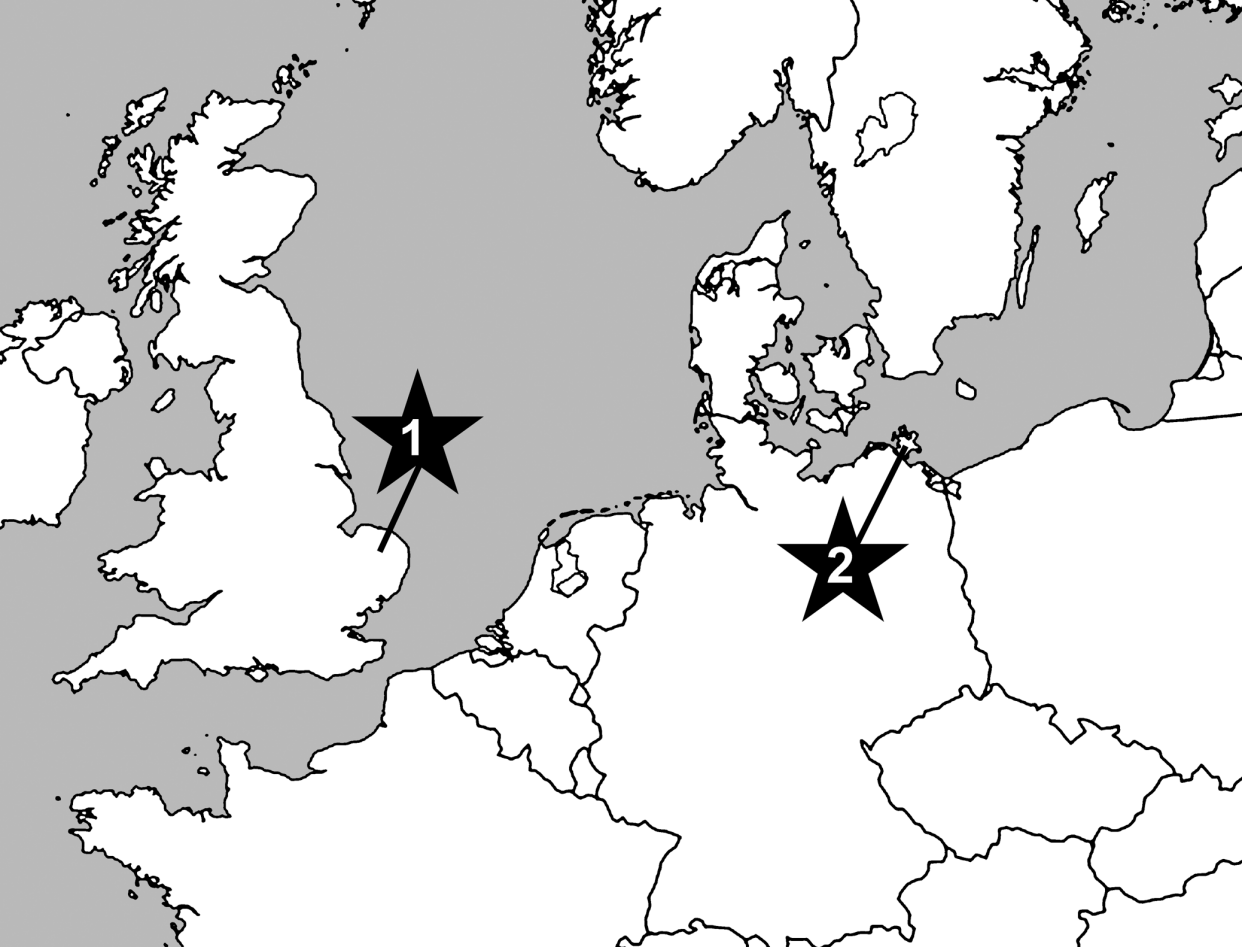
Map showing position of sampled localities at Cringleford near Norwich, Norfolk, United Kingdom (1) and the Isle of Rügen, northeast Germany (2).

**Figure 2:**
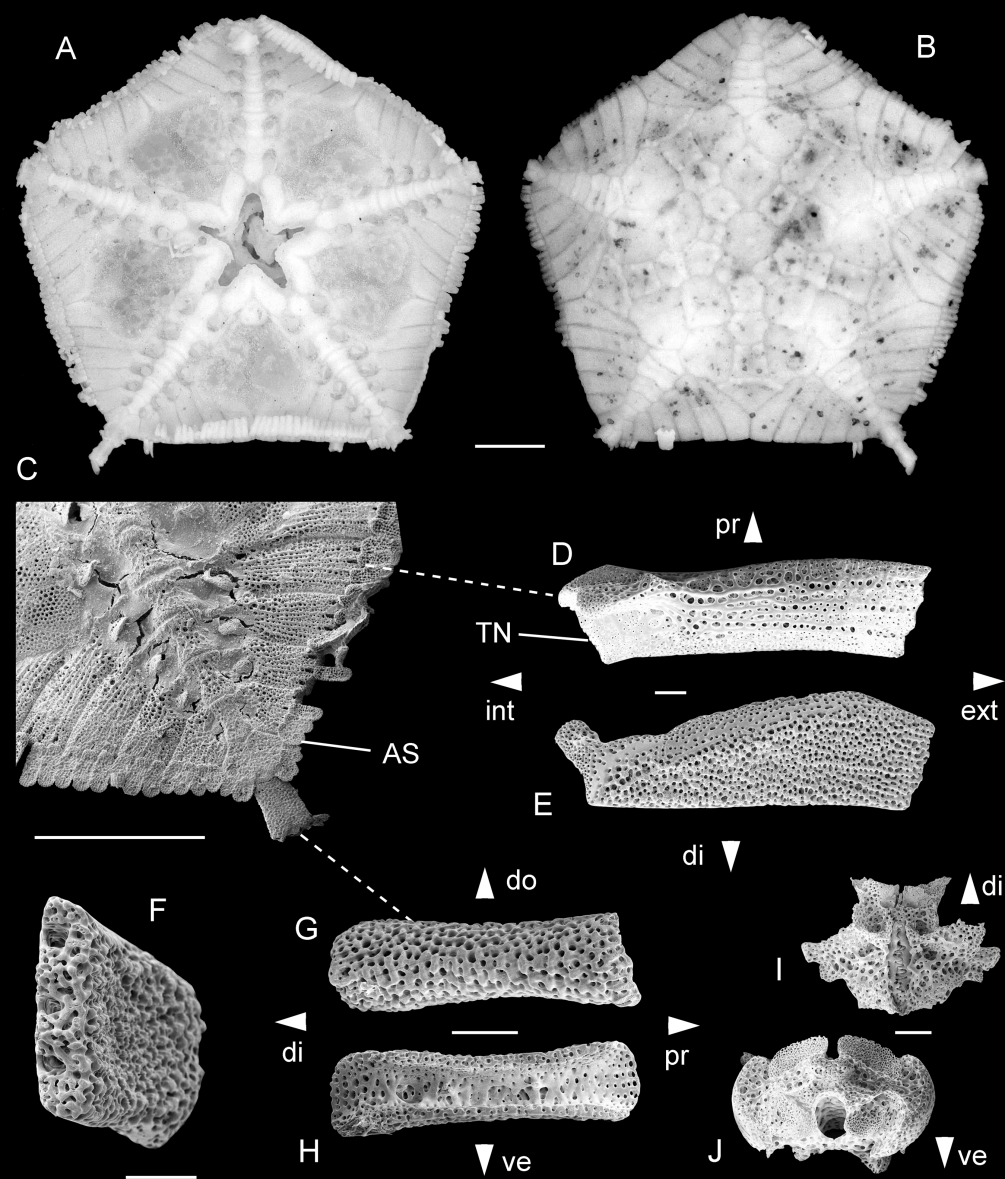
*Astrophiura permira*, Recent, off Natal, South Africa. Complete skeleton in dorsal (A) and ventral (B) aspects; detail of radius in ventral view (C); modified proximal lateral arm plate in ventral (D) and dorsal (E) views, with anatomical position within articulated animal (C) indicated by dashed line; detail of spine articulations (left) in external view (F); regular lateral arm plate in external (G) and internal (H) views, with anatomical position within articulated animal (C) indicated by dashed line; proximal vertebra in ventral (I) and distal (J) views.Abbreviations: AS: arm spine; di: distal; do: dorsal; ext: external; int: internal; pr: proximal; TN: tentacle notch; ve: ventral. Scale bars equal 1 mm (A−C) and 0.1 mm (D−J).

Against all odds, we have now managed to identify fossils from the uppermost Cretaceous of Germany and the UK (Fig. 1) that are unambiguously assignable to the Astrophiuridae. Remarkably analogous to the tale of the discovery of the first living *Astrophiura*, the fossils described herein were initially misidentified as asteroid microfossils until one of us (AG) noted similarities to the lateral arm plates of extant *Astrophiura* (Fig. 2). However, the full impact of this discovery came only after the first scanning electron microscope sessions during which some of the fossil lateral arm plates that had initially been assigned to *Astrophiura* turned out to differ fundamentally with respect to the morphology of their spine articulations, showing patterns typically found in members of the ophionereidoid family Amphilimnidae. Until now, the extant genus *Amphilimna* was the only member of the Amphilimnidae, a clade recently identified as family level taxon (O’Hara et al., 2017). Our discovery adds a second, highly unusual genus to the family. Furthermore, since only a single fossil amphilimnid species was known until now (Thuy, Numberger-Thuy & Jagt, 2018), it expands the fossil record of the family significantly.

Here, we describe the first fossil astrophiurid and an extinct amphilimnid with similarly transformed lateral arm plates, illustrating a remarkable case of parallel evolution.

## Materials and Methods

The material described herein consists of dissociated microfossils picked from residues of screen-washed chalk samples. Most of the specimens are from the white chalk facies of late Early Maastrichtian age (*sumensis*, *cimbrica* and *fastigata* belemnite zones) of the Isle of Rügen, northeast Germany (Fig. 1), handpicked by Manfred Kutscher (Sassnitz, Rügen) from the same samples that yielded the material described and illustrated by Kutscher & Jagt (2000). Two additional specimens were picked by one of us (AG) from the pre-Weybourne Chalk (horizon of the echinoid *Echinocorys subconicula*, *Belemnitella mucronata* belemnite Zone) at Cringleford near Norwich Norfolk, UK (Fig. 1). Sample processing involved simple screen washing with water for the Rügen material. The Cringleford sample was disintegrated using Glauber’s Salt technique (Wissing & Herrig, 1999) before screen-washing.

Deposition of the chalk sediments at both sampled localities took place in an open shelf setting well below storm-wave base, equivalent to a modern deep shelf to shallow slope environment, at the Isle of Rügen (Neumann, 2012; Thuy, 2013) and a deep shelf environment at Cringleford (Wood, 1988). Thus, thee paleo-depth at both localities is reconstructed as shallow bathyal and deep sublittoral, respectively (Neumann, 2012).

Selected specimens were cleaned in an ultrasonic bath, mounted on aluminum stubs and gold-coated for scanning electron microscopy (SEM) using a Jeol Neoscope JCM-5000. Recent specimens of *Astrophiura permira* from the shallow bathyal environment off Natal, South Africa, deposited in the collections of the Natural History Museum, London (collection number NHMUK 1976.7.28.2-8) were examined for comparison. In order to extract relevant skeletal plates, one of these individuals was macerated in household bleach. The dissociated skeletal plates were then rinsed using tap water and mounted on stubs for SEM analysis. All fossil specimens figured herein are now deposited in the collections of the Natural History Museum Luxembourg (acronym MnhnL OPH). Terminology follows Thuy & Stöh (2011, 2016) and Stöhr, O’Hara & Thuy (2012). We adopt the classification by O’Hara et al. (2017, 2018).

The electronic version of this article in Portable Document Format will represent a published work according to the International Commission on Zoological Nomenclature (ICZN), and hence the new names contained in the electronic version are effectively published under that Code from the electronic edition alone. This published work and the nomenclatural acts it contains have been registered in ZooBank, the online registration system for the ICZN. The ZooBank Life Science Identifiers (LSIDs) can be resolved and the associated information viewed through any standard web browser by appending the LSID to the prefix http://zoobank.org/. The LSID for this publication is: urn:lsid:zoobank.org:pub:9619E9BE-36AF-4BC3-9202-4178CF89C028. The online version of this work is archived and available from the following digital repositories: PeerJ, PubMed Central and CLOCKSS.

## Results

Systematic palaeontology

Class Ophiuroidea Gray, 1840

Subclass Myophiuroidea Matsumoto, 1915

Infraclass Metophiurida Matsumoto, 1913 (crown-group of Ophiuroidea)

Superorder Euryophiurida O’Hara et al., 2017

Order Ophiurida Müller & Troschel, 1840

Suborder Ophiurina Müller & Troschel, 1840 sensu O’Hara et al., 2017

Family Astrophiuridae Sladen, 1879

Genus *Astrophiura* Sladen, 1879

**Type species:** *Astrophiura permira* Sladen, 1879, by original designation.

**Diagnosis:** Astrophiurid with large, fragile, limpet-like disc composed distally of a tightly interlocking array of strongly modified, dorso-ventrally flattened lateral arm plates of the proximal arm segments with disproportionately large tentacle pores and arm spines in a horizontal row forming a fringe around the disc edge; regular, slender and elongated arm segments beyond disc edge.

**Etymology:** Species named in honour of Dr. Mark Benecke, German forensic biologist, in recognition of his efforts to promote scientific culture and in particular to encourage both experts and lay people to look for the unexpected and inconspicuous.

**Holotype:** MnhnL OPH078, a dissociated lateral arm plate.

**Type locality and stratum:** The Isle of Rügen, northeast Germany; white chalk facies of late Early Maastrichtian age (*sumensis*, *cimbrica* and *fastigata* belemnite zones).

**Paratypes:** Two dissociated lateral arm plates, MnhnL OPH079 and OPH080.

**Other material studied:** Additional dissociated lateral arm plates, MnhnL OPH081 and OPH082.

**Diagnosis:** Species of *Astrophiura* with stout proximal lateral arm plates covered ventrally by large tubercles and dorsally by both large and smaller tubercles and with up to five spine articulations composed of small muscle and nerve openings separated by a large knob.

**Figure 3:**
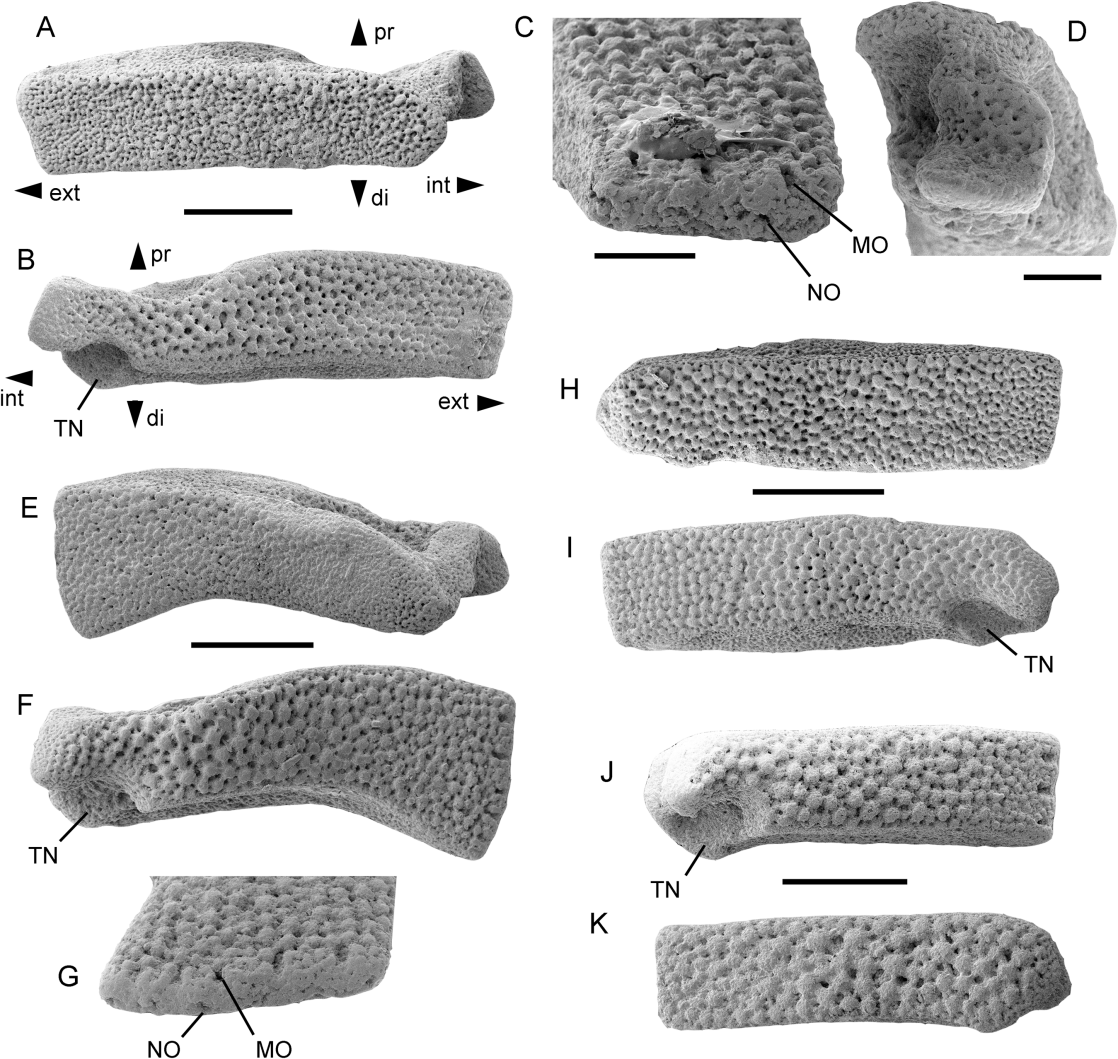
*Astrophiura markbeneckei* sp. nov., dissociated lateral arm plates from the upper lower Maastrichtian of Rügen, Germany (A–I) and from the upper Campanian of Cringleford near Norwich, Norfolk, United Kingdom (J, K). MnhnL OPH078 (holotype) in dorsal (A) and ventral (B) views, and details of spine articulations (C) and of inner side (D). MnhnL OPH079 (paratype) in dorsal (E) and ventral (F) views, and detail of spine artriculations (G). MnhnL OPH080 (paratype) in dorsal (H) and ventral (I) views. MnhnL OPH082 in ventral (J) and dorsal (K) views. Abbreviations: di: distal; do: dorsal; ext: external; int: internal; MO: muscle opening; NO: nerve opening; pr: proximal; TN: tentacle notch; ve: ventral. Scale bars equal 0.5 mm (A,B, E, F, H−K) and 0.2 mm (C, D, G).

**Description of holotype:** MnhnL OPH078 (Figs. 3A−3D) is a dissociated proximal lateral arm plate, extremely widened and dorso-ventrally flattened; outer surface tapered to a horizontal, very thin edge carrying three spine articulations in a row; distal and proximal edges of the lateral arm plate transformed into vertical contact surfaces with neighbouring lateral arm plates; ventral surface of lateral arm plate covered by large, roughly similarly sized tubercles surrounded by stereom pores of two different sizes, the larger ones almost the size of the tubercles, the other ones one third smaller; dorsal surface of the lateral arm plate covered by tubercles of various sizes, the largest ones of the same size as those on the ventral side, the smaller ones approximately half the size, with small, roughly similarly sized stereom pores surrounding the tubercles; inner side with single, large ridge articulating with the associated vertebra; ventro-distal edge of the lateral arm plate with a large, conspicuous tentacle notch; ventro-proximal edge concave; three spine articulations of same size, oblique, extending on the ventral side of the lateral arm plate, composed of a small muscle opening separated from a slightly smaller nerve opening by a large, conspicuous knob.

**Paratype supplements and variation:** MnhnL OPH079 (Figs. 3E−3G) is a dissociated proximal lateral arm plate similar to the holotype but with a slightly wider and curved distal half; ornamentation on dorsal and ventral surfaces as in holotype; five spine articulations on tapered horizontal edge of outer surface.

MnhnL OPH080 (Figs. 3H−3I) is a dissociated proximal lateral arm plate similar to holotype but with strait ventro-proximal edge.

**Remarks:** The lateral arm plates described above differ from any other type of lateral arm plate ever described from the fossil record, but they do perfectly match the lateral arm plates illustrated by Fujita & Hendler (2001) extracted from specimens of their new extant species, *Astrophiura wanikawa* Fujita & Hendler (2001). We have macerated a specimen of the type species *Astrophiura permira* and confirmed the similarities to our fossils. In no other currently known ophiuroid genus are the lateral arm plates so strongly modified. Proximal arm joints of the astrophiurid genera *Ophiomisidium* and *Ophiophycis* may have enlarged lateral arm plates as well, in some cases even forming a similarly tightly interlocking extension of the disc (e.g. in *Ophiophycis guillei* Vadon, 1991) but they are never as elongate as in *Astrophiura*. We therefore assign the fossils described herein to *Astrophiura*.

The genus currently includes nine extant species which differ only by few and often subtle diagnostic characters (Fujita & Hendler, 2001; Stöhr, O’Hara & Thuy, 2012). To make matters worse, descriptions of living ophiuroid species often lack data on the microstructural details needed for a conclusive comparison with fossil dissociated lateral arm plates. A brief revision of descriptions of modern species of *Astrophiura* suggests that none has proximal lateral arm plates as stout and as strongly tuberculated as the fossils described herein. Furthermore, living species of *Astrophiura* tend to have fewer and smaller arm spine articulations. Therefore, and in addition to an implausibly long stratigraphic range for a living species, we assign the Late Cretaceous fossils described herein to a new species.

**Occurrence:** Upper lower Maastrichtian of Rügen, northeast Germany, and upper Campanian of Cringleford near Norwich, UK (Figs. 3J−3K).

Superorder Ophintegrida O’Hara et al., 2017

Order Amphilepidida O’Hara et al., 2017

Suborder Ophionereidina O’Hara et al., 2017

Superfamily Ophionereidoidea Ljungman, 1867

Family Amphilimnidae O’Hara et al., 2018

Genus *Astrosombra* nov.

**Type species:** *Astrosombra rammsteinensis* sp. nov., by present designation.

**Diagnosis:** Amphilimnid with strongly modified lateral arm plates, dorso-ventrally flattened, with oblique distal and ventral edges probably allowing for overlap of neighbouring lateral arm plates, with coarsely tuberculated outer surface ornamentation, and with near-horizontal row of spine articulations lining the external edge of the lateral arm plate, with the proximal (originally dorsal) spine articulations small and composed of thin, close-set ridges and the distal (originally ventral) spine articulations much larger and composed of thicker, widely spaced ridges.

**Etymology:** Name formed by combining ‘Astro’, derived from the Greek ‘astron’ (meaning celestrial body) and referring to the superficial similarity with the non-related, star-shaped *Astrophiura*, and ‘sombra’, in reference to the Spanish word ‘sombra’ (meaning shade) and the French word ‘sombre’ (meaning dark), thus collectively translating into ‘dark star’.

**Gender:** feminine.

**Etymology:** Species named in honour of German rock band Rammstein, in recognition of their musical achievements, and because they are true dark stars.

**Holotype:** MnhnL OPH083, a dissociated lateral arm plate.

**Type locality and stratum:** The Isle of Rügen, northeast Germany; white chalk facies of late Early Maastrichtian age (*sumensis*, *cimbrica* and *fastigata* belemnite zones).

**Paratypes:** Tow dissociated lateral arm plates, MnhnL OPH084 and OPH085.

**Other material studied:** Additional dissociated lateral arm plates MnhnL OPH086.

**Diagnosis:** as for genus.

**Figure 4:**
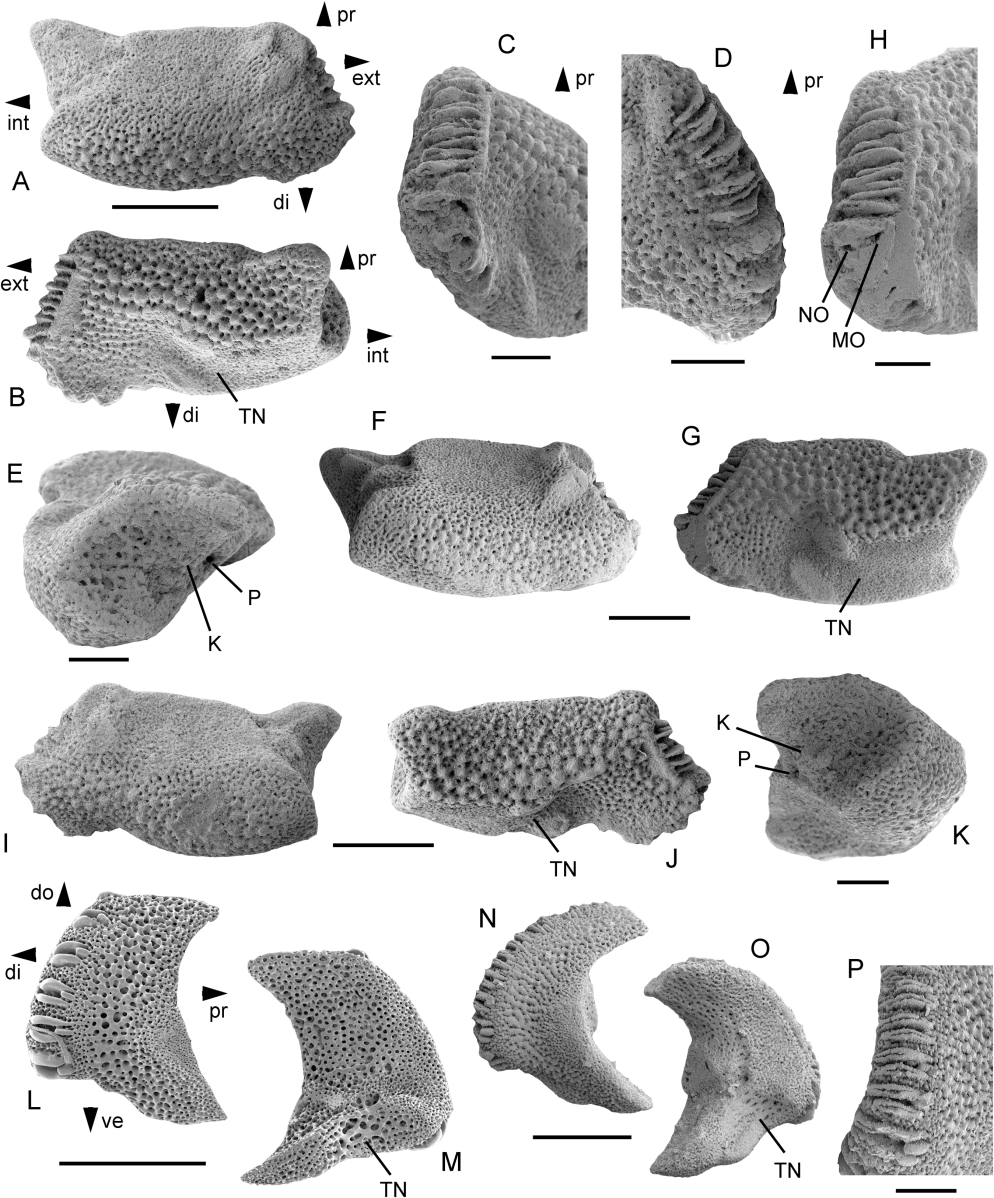
*Astrosombra rammsteinensis* gen. et sp. nov., dissociated lateral arm plates from the upper lower Maastrichtian of Rügen, Germany. MnhnL OPH083 (holotype) in dorsal (A) and ventral (B) views, with details of distal (originally ventral) (C) and proximal (originally dorsal) (D) arm spine articulations, and with detail of inner side (E). MnhnL OPH084 (paratype) in dorsal (F) and ventral (G) views, with detail of spine articulations (H). MnhnL OPH085 (paratype) in dorsal (I) and ventral (J) views, with detail of inner side (K). Dissociated proximal lateral arm plate of *Amphilimna olivacea*, Recent, in external (L) and internal (M) views, modified from Thuy et al. (2014). Dissociated proximal lateral arm plate of *Amphilimna intersepultosetme*, Maastrichtian of South Carolina, in external (N) and internal (O) views and with detail of spine articulations (P), modified from Thuy, Numberger-Thuy & Jagt (2018). Abbreviations: di: distal; do: dorsal; ext: external; int: internal; K: knob for vertebral articulation; MO: muscle opening; NO: nerve opening; P: perforation; pr: proximal; TN: tentacle notch; ve: ventral. Scale bars equal 0.5 mm (A, B, F, G, I, J, L−O) and 0.2 mm (C−E, H, K, P).

**Description of holotype:** MnhnL OPH083 (Figs. 4A−4E) is a dissociated proximal lateral arm plate, extremely widened and dorso-ventrally flattened; outer surface tapered to a horizontal, thin edge carrying eight spine articulations in a continuous but heterogeneous row; proximal edge of the lateral arm plate transformed into oblique contact surface with neighbouring lateral arm plates, with rounded transitions to dorsal and ventral outer surface and with two moderately well-defined, oval, prominent spurs composed of more densely meshed stereom and located on the outermost edge; distal edge of the lateral arm plate transformed into a similarly oblique contact surface with two spurs similar to those on proximal edge surface but located closer towards the midline of the lateral arm plate; ventral and dorsal outer surfaces of lateral arm plate covered by large, rounded tubercles of varying size, largest in the centre, gradually decreasing to half the size towards the distal edge of the ventral outer surface and to one-third of the size towards the proximal edge of the outer surface; inner side with a single well-defined perforation and a very weakly discernible single vertebral articulation knob; ventro-distal edge of the lateral arm plate with a very large, conspicuous tentacle notch, poorly defined, covered by small tubercles and surrounded by spine articulations towards the outer edge, and sharply defined and bordered by a prominent knob towards the inner edge; eight spine articulations in total, arranged in a continuous horizontal row along outer edge of the lateral arm plate; the four proximal (originally dorsal) spine articulations moderately large, closely spaced, composed of very long, thin, parallel lobes, a double ventral one and a simple dorsal one, nerve and muscle openings not distinguishable; four dorsal spine articulations with a proximalward (originally dorsalward) decrease in size and ventrally (originally distally) bordered by a sharp ridge; four distal (originally ventral) spine articulations much larger, of similar sized, with a distalward (originally ventralward) increase in size of gap between spine articulations; dorsal and ventral lobes thicker, parallel, and dorsally (originally proximally) separated by two to three short, small knobs; muscle and nerve openings of similar size.

**Paratype supplements and variation:** MnhnL OPH084 (Figs. 4F−4H) is a dissociated proximal lateral arm plate generally similar to the holotype but slightly longer and with the distal (originally ventral) spine articulations not protruding distalwards as strongly as in the holotype.

MnhnL OPH085 (Figs. 4I−4K) is a dissociated median (?) lateral arm plate; general morphology as in the holotype but with slightly more strongly convex dorso-distal edge; seven spine articulations comprised of three smaller proximal (originally dorsal) ones and four larger distal (originally ventral) ones; single knob on inner side of lateral arm plate slightly better visible than in the holotype.

**Remarks:** Due to striking similarities in general shape and outer surface ornamentation, the lateral arm plates described above were initially mistaken for remains of the co-occurring *Astrophiura markbeneckei* sp. nov. SEM, however, later revealed fundamental differences in the structure of the spine articulations. While *Astrophiura* shows the typical ophiurid spine articulation composed of nerve and muscle openings widely separated by a large knob but lacking dorsal and ventral lobes, the lateral arm plates in question have conspicuous dorsal and ventral lobes associated in straight and parallel pairs and separated proximally by one or several small, elongate knobs, as typically seen in the family Amphilimnidae. Lateral arm plates of the extant *Ophiopsila*, the only known genus of the family Ophiopsilidae (O’Hara et al., 2018), have superficially similar spine articulations (Thuy & Stöhr, 2016). A more detailed comparison, however, reveals that the lateral arm plates described herein show longitudinally divided dorsal and ventral lobes, strikingly similar to those observed on the lateral arm plates of *Amphilimna intersepultosetme* Thuy, Numberger-Thuy & Jagt (2018) from the uppermost Cretaceous of South Carolina. We therefore assign the specimens described above to the Amphilimnidae.

The only known genus of the family (*Amphilimna*), however, has sickle-shaped lateral arm plates (Thuy, Numberger-Thuy & Jagt, 2018) that fundamentally differ from the hypertrophied, dorso-ventrally flattened ones described above. Given the pivotal diagnostic value of the spine articulation morphology in ophiuroid phylogeny (Thuy & Stöhr, 2016), we assign these lateral arm plates to the family Amphilimnidae and introduce a new taxon, *Astrosombra rammsteinensis*, as its second known genus.

In consequence, the hypertrophism and dorso-ventral flattening of the lateral arm plates are to be considered as cases of convergent evolution with respect to *Astrophiura*. Although *Astrosombra* is currently known only from dissociated lateral arm plates, it seems highly probable that the latter formed a tightly interlocking extension of the disc, similar to those of *Astrophiura*. However, the oblique rather than straight contact surfaces with the neighbouring lateral arm plates are different; this results in a tile-like overlapping of the plates.

**Occurrence:** Upper lower Maastrichtian of Rügen, Germany.

## Discussion

The fossils described herein significantly add to our knowledge of ophiuroid evolutionary history. First, *A. markbeneckei* sp. nov. represents the first example from the fossil record of the Astrophiuridae, bridging at least part of the exceedingly long ghost lineage that separate modern members of the family from its assumed Early Jurassic origin (O’Hara et al., 2017). Second, the new taxon *Astrosombra rammsteinensis* gen. et sp. nov. is only the second genus of the family Amphilimnidae and significantly expands the morphological spectrum of the latter. It also shows that the peculiar morphological modifications of *Astrophiura*, resulting in a pentagonal starfish- or limpet-like body shape, has evolved independently in at least two unrelated ophiuroid families.

The majority of remains of *A. markbeneckei* sp. nov. were recovered from the white chalk facies of Rügen, deposited at shallow bathyal depths (Thuy, 2013). Thus, from a palaeoecological point of view, our discovery suggests that the Astrophiuridae already inhabited bathyal environments during the Late Cretaceous. Modern astrophiurids commonly occur on hard substrates (rocks, empty shells or even beer bottles) in the deep sea (Fujita & Hendler, 2001; Pawson, 2018). The morphological modifications of the skeleton entail an increased ventral body surface and strongly enlarged tube feet, thus facilitating adherence to hard substrates (Fujita & Hendler, 2001). Given a similar level of hypertrophism and dorso-ventral flattening and similarly large tentacle openings in both *A. markbeneckei* sp. nov. and *Astrosombra rammsteinensis* gen. et sp. nov., with respect to living astrophiurids, we can assume that they had similar functional adaptations to living on hard substrates.

## Conclusions

The family Astrophiuridae has recently been identified as a family-level clade within the ophiuroid order Ophiurida. In spite of its assumed ancient age, the family was completely absent from the fossil record until now. Using dissociated skeletal plates preserved as microfossils from sediments dated as latest Cretaceous, we have identified the first fossil occurrences of the Astrophiuridae and assigned them to the new species *A. markbeneckei* sp. nov. The geological background of the new fossils, preserved in shallow bathyal sedimentary rocks, suggests that the Astrophiuridae have inhabited the deep sea at least since the latest Cretaceous. Our material has turned out to yield remains of an additional taxon, *Astrosombra rammsteinensis* gen. et sp. nov., which is superficially similar to *A. markbeneckei* sp. nov., but is assignable to a different family Amphilimnidae on account of spine articulation morphology. This suggests that skeletal modifications leading to a pentagonal starfish-like body shape in ophiuroids have evolved independently at least twice. Finally, our discoveries demonstrate once more that the inclusion of microfossils significantly adds to our understanding of the evolutionary history of taxa with multi-element skeletons such as ophiuroids.

## Additional Information and Declarations

### Data Availability

The following information was supplied regarding data availability: Data is available at Figshare: Thuy, Ben (2019): Raw data for article “Brittle stars looking like starfish: the first fossil record of the Astrophiuridae and a remarkable case of convergent evolution”. figshare. Figure. DOI 10.6084/m9.figshare.9919052.v1 Accession numbers of the mentioned specimens: MnhnL OPH078 - OPH086 are housed at the Luxembourg Natural History Museum, and NHMUK 1976.7.28.2-8 are housed at the Natural History Museum, London.

### New Species Registration

The following information was supplied regarding the registration of a newly described species: Publication LSID: urn:lsid:zoobank.org:pub:9619E9BE-36AF-4BC3-9202-4178CF89C028. Astrosombra LSID: urn:lsid:zoobank.org:act:2B81D361-F1D1-4368-A7F2-EC58758B943C. Astrosombra rammsteinensis LSID: urn:lsid:zoobank.org:act:D55E0270-F3FE-4A5A-9824-2F6BD7DBE4E0. *Astrophiura markbeneckei* LSID: urn:lsid:zoobank.org:act:267A18F3-0A7A-4427-A683-076B05F6EA11.
